# Toward process-based causality indicators for assessing and monitoring ecological impacts of offshore wind energy: a benthic perspective

**DOI:** 10.1007/s10661-026-15734-y

**Published:** 2026-07-25

**Authors:** Ivan Rodrigo Abrão Laurino, Anna Thereza dos Santos Alves Silva, Bruno Olian, Márcia Regina Denadai, Luis Enrique Sánchez, Alexander Turra

**Affiliations:** 1 Laboratory of Management, Ecology and Marine Conservation, Oceanographic Institute (IO-USP), University of Sao Paulo, Praça Oceanográfico, 191 Butantã, São Paulo, SP 05508-120 Brazil; 2 Power Systems Innovation Hub (RCGI-InnovaPower), University of Sao Paulo, Av. Prof. Luciano Gualberto, 380 Butantã, São Paulo, SP 05508-010 Brazil; 3 Escola Politécnica, University of Sao Paulo, Av. Prof. Mello Moraes, 2373 Butantã, São Paulo, SP 05508-900 Brazil; 4 Institute of Advanced Studies (IEA-USP), University of Sao Paulo, Rua da Praça Do Relógio, 109, Butantã, São Paulo, SP 05508-050 Brazil

**Keywords:** Renewable energy, Life-history traits, Causal chain, Cumulative effects, EIA

## Abstract

Offshore wind energy (OWE) is rapidly emerging and expanding in several countries worldwide. A current challenge is understanding the ecological-causal processes underlying OWE environmental impacts to determine appropriate indicators for baseline assessment and follow-up monitoring. Here, we reviewed the ecological impacts and screened parameters that can serve as process-based indicators of causality for assessing and monitoring changes in benthic environments associated with OWE development. To this end, we first conducted a global literature review (73 articles) to compile evidence on OWE impacts on benthic environments. Next, based on the assessed evidence, we designed conceptual-qualitative ecosystem models to clarify the ecological-causal processes connecting OWE pressures with their potential cascading effects. Lastly, we identified ecological parameters from the reviewed literature that could serve as indicators of the processes described by the models. As key results, we detailed the causal chain of OWE effects on benthic environments and proposed 32 indicators for assessing and monitoring impacts. We highlighted that the causal meaning of the indicators, from a process-based perspective, depends on considering biodiversity categorized by life-history traits, rather than relying on general parameters such as the total abundance of the benthic community. This includes classifying species into ecological functional groups that will signal distinct causal processes, such as noise pollution, organic enrichment, and changes in ecosystem functioning. Such a tool can be further applied in baseline and monitoring studies to guide Environmental Impact Assessment toward a process-based praxis that is more aligned with the principles of Ecosystem-Based Management.

**Supplementary Information:**

The online version contains supplementary material available at 10.1007/s10661-026-15734-y.

## Introduction

Climate change is one of the most significant challenges facing humankind in the twenty-first century (IPBES, 2019), with the transition to a low-carbon economy being a primary means of addressing its consequences (IPCC, 2023). Advancing renewable energy production is crucial in this context, and wind energy has emerged globally as a key alternative (Nguyen et al., 2024). However, like any industrial development, wind farms use natural resources and interact with biodiversity and socioecological systems, thereby affecting various ecological processes, species, habitats, ecosystem services, and nearby human populations (Leung & Yang, 2012; Watson et al., 2024). Offshore wind energy (OWE) production emerged at the end of the last century to harness vast wind resources while potentially avoiding the harmful impacts of onshore wind farms, albeit introducing new ecological challenges associated with its interactions with the marine environment (Hernandez et al., 2021; Kaldellis et al., 2016). Assessing and monitoring the effects of OWE projects on vulnerable marine environments, including associated biodiversity and the benefits they provide to people, are essential to support management, planning, and mitigation strategies, thereby ensuring more sustainable production (Abramic et al., 2022; Siqueira-Gay et al., 2025).

Benthic environments are among the primary receptors of impacts from OWE development, as turbines, power transmission cables, and support substations interact directly with the seabed (Dannheim et al., 2020; Stranddorf et al., 2025). Both the installation and operation of these projects significantly affect benthic ecosystem functioning, as artificial hard structures are typically introduced into areas previously dominated by soft substrates (sediments) (Dannheim et al., 2020; Degraer et al., 2020). Benthic ecosystems are recognized as crucial for marine biodiversity, as they can host hotspots of biological diversity and species vital to providing ecosystem services and resources for both artisanal and industrial fisheries (Crespo & Pardal, 2022). Consequently, an effective Environmental Impact Assessment (EIA) should include benthic ecosystems, aiming to identify which components and ecological properties of the system (i.e., processes) are vulnerable to the various activities and pressures of the OWE projects.

In the EIA context, developing a baseline is essential to characterize and understand the environmental attributes that can be affected by a new OWE project, ensuring that the reference condition (before project influence) of priority ecological processes and components, including benthic habitats and communities, is accurately measured (e.g., Laurino et al., 2025; Tamis et al., 2021). To achieve this goal, key parameters of the different components and processes must be selected for assessment in the baseline phase. Later, these same parameters can be monitored as indicators to identify predicted (or non-predicted) changes, compared to baseline values, resulting from the project’s development (e.g., Methratta, 2024; Taormina et al., 2022; Trifonova et al., 2021). This implies that the selection of indicators for impact assessment and monitoring must be made early in the EIA process and be precise to effectively support the identification of the causality of OWE impacts (Laurino et al., 2025).

The development of an incomplete or poorly focused baseline can hinder EIA and ultimately lead to unpredicted significant impacts (Clark et al., 2020; Dias et al., 2022). It is common for a large amount of data to be sampled to assess a project’s impacts, but without a clear purpose (i.e., “what for”), such data can translate into little useful information, particularly when they cannot demonstrate cause-and-effect relationships to support interpretations regarding the impacts on ecosystem functioning (Wilding et al., 2017). This may also hinder the identification of appropriate mitigation measures., This issue persists worldwide because indicators are not selected based on the ecological-causal processes that link OWE pressures to their cascading environmental effects (Laurino et al., 2025). Under such a “data-rich, information-poor” scenario (Wilding et al., 2017), baseline assessment generates unnecessary costs and provides little (if any) assistance in the EIA process. This problem underscores the need for studies that help establish process-based causality indicators, represented by meaningful metrics derived from the ecological-causal processes of the OWE project (Laurino et al., 2025). Such indicators are useful because they can be sampled during the baseline phase to support cause-and-effect interpretations and the establishment of ecological thresholds for monitoring (Han et al., 2025).

Ecosystemic approaches that value a holistic, integrated view of ecological processes in the environment can contribute to more effective indicator selection for EIA (Andrade & Turra, 2021; Laurino et al., 2025; Turra et al., 2017). In this context, the principles of Ecosystem-Based Management (EBM) highlight important insights to follow (e.g., Guilhon et al., 2021, 2023), including consideration of the dynamics of system components, their connections, and potential cumulative properties (Long et al., 2015). In terms of the EIA, this means considering the processes sustaining the components and their relationships, including trophic connections between species, and even how pressures caused by environmental changes act additively or synergistically along a causal chain of events (Perdicoúlis & Piper, 2008; Raoux et al., 2017, 2019; Wilding et al., 2017). By incorporating EBM principles, parameters such as species richness or the abundance of a given organism gain process-based meaning and can serve as indicators within the causal nexus between the project and the environment (Bravo et al., 2023; Wilding et al., 2017). Changes in these indicators detected during monitoring can thus indicate impacts in processes with clear connections within the causal chain (Bravo et al., 2023; Causon & Gill, 2018), ultimately clarifying the effects on ecosystem services (Baulaz et al., 2023; Van de Pol et al., 2023).

The construction of a conceptual ecosystem model serves as a valuable EBM tool to assess the ecological-causal processes linking the pressures of a project and the respective environmental components affected (physical, biotic, and socioeconomic), as well as their ecosystemic cascading consequences (e.g., Tamis et al., 2021; Baulaz et al., 2023; Van de Pol et al., 2023). This approach enables us to understand in advance, based on information collected from previous experiences, how a given component attribute may vary qualitatively (i.e., increase or decrease) in response to a particular pressure (Perdicoúlis & Glasson, 2006; Perdicoúlis & Piper, 2008). Conceptual qualitative models are simple and easy to design because they are based solely on prior evidence to support general patterns of causality, connections, and consequences, and/or a general understanding of natural processes (Laurino et al., 2025). At the same time, such an approach is highly useful as a first step to suggesting causality processes, supporting fieldwork guidelines to later design quantitative models in the local context for practical applicability in the EIA (e.g., Raoux et al., 2017; Trifonova & Scott, 2023; Trifonova et al., 2021). Once the causal nexus is clear, it is possible to identify process-based parameters that can serve as indicators of expected changes, thereby directing baseline sampling and monitoring to data with causal meaning (Bravo et al., 2023; Trifonova & Scott, 2023; Wilding et al., 2017).

In this study, we use these EBM conceptual models to identify which ecological parameters would be meaningful process-based indicators of causality for assessing and monitoring changes in benthic environments related to the installation and operation of OWE projects. Specifically, we first conducted a bibliographic review of the major known effects of OWE in benthic environments, their impacts on biodiversity, and associated ecological processes. Secondly, based on the evidence collected in the review, we designed conceptual-qualitative ecosystem models that clarify the ecological-causal processes linking the identified project pressures to their potential cascading consequences in the benthic environment. Lastly, we identified which ecological parameters present in the analyzed bibliography could serve as useful indicators for capturing the changes associated with the ecological-causal processes highlighted by the models. In this last step, to exemplify the application of the proposed indicators, we also developed scenarios of indicator responses to suggest distinct hypotheses of ecological effects that would be raised by indicator variations during monitoring of the impacts of OWE projects. Thus, our purpose was to improve the impact assessment of OWE projects by unraveling parameters to be assessed during the baseline phase and later be used as process-based causality indicators during the monitoring phase.

## Materials and methods

### Bibliographic review

A bibliographic survey of original research articles was conducted to understand the major known effects of OWE in benthic environments. Two academic platforms were used for the search: Scopus and Web of Science. The same search string was applied for both platforms, including keywords such as “offshore wind,” “impact,” “effect,” “benthic,” “bottom,” “ecosystem,” “biodiversity,” among others (see supplementary material, Appendix A, for details about the search string). The results were then narrowed to original research papers written in English, with no temporal restrictions up to the search date (October 2024). A total of 302 results were obtained (139 from Scopus and 163 from Web of Science). After excluding the duplicates (124 articles), a total of 178 articles remained for the screening phase.

As our specific goal was to gather the best evidence of effects of OWE in the benthic environment, we were primarily interested in case studies from around the world that reported clear, specific effects. Therefore, during the screening phase, only those articles that fit our needs were selected using four exclusion criteria: (1) the article was not an original research article (i.e., it was a review article, conference paper, book chapter, technical report, data article without tests, or methodological articles); (2) the article was unrelated to the installation and/or operation of OWE projects or their associated activities (such as dredging and pile-driving); (3) the article was unrelated to benthic environments and/or their biodiversity; (4) the article did not aim to explore and test possible OWE effects (including their associated activities) on benthic environments and/or their biodiversity. Modeling studies using field data based on installed and operating OWE undertakings (not future projects) were considered. We also considered articles reporting results from field and lab experiments simulating impacts associated with OWE projects and related activities. The screening process began with the reading of the titles, abstracts, and keywords of all 178 articles found. Articles that met at least one of our exclusion criteria were removed from the list during this process, and, in cases of doubt, a full reading of the article was required before the decision. In total, 114 articles were excluded, and 64 were selected for the next phase.

To complement our survey and ensure that all major impacts of OWE projects in benthic environments were included, a second phase of the search was conducted, seeking original research articles cited by two recent reviews: Dannheim et al. (2020) and Watson et al. (2024). Both reviews provide comprehensive supplementary materials, including several original research papers on the environmental impacts of offshore renewables. Specifically, Dannheim et al. (2020) focused on the benthic effects of offshore renewables, drawing on a vast bibliography spanning many types of offshore projects, including OWE projects. Watson et al. (2024) focused on the impacts of OWE on ecosystem services, including many articles regarding the benthic environment. These reviews and their respective supplementary materials were analyzed to identify potential effects not included in the 64 previously screened articles. During this analysis, when we found an effect not considered in the first phase, we identified the original reference cited by the review authors and considered adding it to our final list of articles. Such consideration was made based on the same exclusion criteria described before. Therefore, only original research articles, testing the effects of OWE in benthic environments and species, were selected from the cited references in these reviews. Nine additional articles were selected in this second search phase and added to our final list, which comprised 73 original research articles in total (see the supplementary material, Appendix A, for more details). Lastly, the articles were classified by thematic topics (the main process subject of the research), components (system elements affected and investigated), and project phases (installation, operation, or both).

### Conceptual ecosystem models

The construction of the conceptual models was based on a full-text analysis of the 73 original research articles identified in our bibliographic review. The model was primarily designed based on the causality analysis and networks described by Perdicoúlis and Glasson (2006) and Perdicoúlis and Piper (2008), which form a framework for environmental impact assessment (see also Turra et al., 2017). We also based the terms and structure of our models partially on the DAPSI(W)R(M) framework (Elliott et al., 2017). Thus, for the model’s construction, the analysis of the articles focused on identifying: (1) the evidenced drivers; (2) the causal system elements (i.e., activities and pressures); (3) the affected system elements (i.e., natural environmental components); and (4) the effects (i.e., state changes in the natural environmental components).

The drivers are defined as the human needs that initiate the cascade of events (Elliott et al., 2017), which, in our models, are represented by wind energy production, divided into two key phases: installation and operation. For each of these drivers (i.e., project phases), we identified the principal activities (i.e., deliberate actions), which are the system elements that represented the primary cause of the observed effects (e.g., pile driving and cable installation). Next, the multiple pressures were identified, which are the environmental aspects (in the sense of the ISO 14001 standard, see Sánchez & Hacking, 2002) generated by the activities in the environment (e.g., underwater sound generation and mechanical shock), also defined as the mechanisms of change in the natural system (Elliott et al., 2017). The set of activities and pressures identified for each driver was considered as the causal system elements of the model (Perdicoúlis & Glasson, 2006).

After identifying these causal system elements, the affected system elements were registered as multiple environmental components (e.g., seabed and infauna) that change state in response to the causal system elements (Perdicoúlis & Glasson, 2006). Although our focus was the benthic environment, we also considered components of the pelagic environment as affected system elements, especially those with a clear connection to the components and processes of the bottom environment (e.g., seawater). Next, all state changes in the affected system elements identified as consequences of causal system elements were defined as effects. Changes that were immediate consequences of the causal elements were classified as direct effects, representing the primary changes caused by the project. Changes that were cascade consequences of the direct effects were classified as indirect effects (including secondary, tertiary, and quaternary changes). For each registered effect, we indicated the direction of the structural relationship between causal and affected elements, labeling changes as “increased” or “decreased.”

The evidenced changes were organized into conceptual diagrams, integrating the results from different articles to illustrate the ecological-causal nexus and the cascade of effects. We used branched tree diagrams (Perdicoúlis & Glasson, 2006), with arrows representing causality, separating the causal chain of the installation and operation phases. The multiple branches in each diagram were numbered and assigned an ID (branch identification). Connections between branches were shown in the diagrams as distinct chains of events that joined along the cascade to reach the same endpoint, indicating cumulative effects. Only causal relationships evidenced by the results of the analyzed articles were included in the models; thus, all branches formed in the diagrams were referenced (see Appendix B of the supplementary material).

### Process-based causality indicators

The survey of potential indicators for assessing the impacts of OWE projects in benthic environments was conducted during the full-text analysis of the articles. For each article, we identified and extracted multiple ecological/biophysical parameters used by the authors to support the observed effects on natural components. It included physical, chemical, or biological metrics related to the affected system elements, measured across the different articles analyzed. Each identified parameter was defined as a potential indicator of the observed effects on the environmental components. Thus, a list of suggested indicators was compiled after the full-text analysis was completed (see supplementary material, Appendix C).

Ecological parameters that we considered as indicators were those directly or indirectly linked to one or more key changes in the ecological-causal nexus observed in the conceptual models. Also, the indicators considered were those parameters that are feasible to sample in the field and usually provide precise, clear responses to changes, based on the results of the articles analyzed. At least one indicator was proposed for each branch formed in the conceptual models. Thus, each branch was considered an ecological-causal process that can be assessed and monitored by the suggested indicator. For more complex branches/processes, multiple indicators were suggested (where available in the analyzed literature) to provide a more complete understanding of the causal nexus.

After identifying the indicators, to exemplify their potential applications, scenarios of indicator responses were proposed, considering the observed behaviors of multiple indicators and suggesting distinct hypotheses of ecological effects that could be raised from an integrated view of the indicators’ variations while monitoring OWE projects. In this step, we focused on key effects expected during the operation phase, as they had the clearest ecological-causal processes established in the analyzed literature (see below). Thus, two integrated response scenarios were designed: one using indicators of the infaunal community (soft-bottom macrofauna) and another using indicators of the epifaunal community (mobile invertebrates and demersal fish). The scenarios included integrated interpretations (hypotheses) of positive and negative effects based on increases or decreases in the selected multiple indicators. Only indicator responses with evidence in the literature analyzed were included in the designed scenarios (all indicator responses were referenced in the supplementary material, Appendix C).

## Results

### Bibliographic review

Among the 73 articles analyzed, 70 tested and found evidence of at least one effect concerning the installation and/or operation of OWE projects in the benthic environment. We identified a total of 14 general thematic topics across the entire literature analysis: five involved the installation and operation phases, while nine were exclusively related to operation (Table 1). The most-studied topic was the reef effect (26 references in total), which explores the colonization of invertebrate and fish species near the project’s artificial structures due to the creation of new hard-bottom habitats. The literature also largely addressed the changes in the composition of the benthic communities (21 references), including infaunal and epifaunal assemblages. The less-studied topics were the release of contaminants (one reference) and changes in primary production (three references). The effects of electromagnetic fields from transmission cables, changes in trophic relationships, and the colonization of non-native species were also understudied (five references each). The system elements affected by the impacts (components) include the seabed (especially soft-bottom habitats), seawater, ecosystem functioning (e.g., nutrient cycling, energy flow), fouling assemblages, and infaunal and epifaunal communities (including mobile invertebrates and demersal fish) (Table 1).

**Table 1:** Synthesis of the general thematic topics found in the literature review on the major effects of the installation and operation of offshore wind projects on the benthic environment, highlighting: the main components (affected system elements); the related phase of the project, including installation (I) and/or operation (O); and the references that addressed each topic

General thematic topic	Components	Project phase	References
Seabed physicochemical changes	Seabed	I and O	De Backer et al. (2014); Coates et al. (2014, 2015); Wang et al. (2018); Lu et al. (2020); Ivanov et al. (2021); Daewel et al. (2022); Mavraki et al. (2022); Guarinello and Carey (2022); Lefaible et al. (2023); Wang et al. (2023)
Changes in hydrodynamics and sediment flow	Seabed; seawater	I and O	Vanhellemont and Ruddick (2014); Wang et al. (2018); Wang et al. (2019); Daewel et al. (2022); Huang (2022); Wang et al. (2023); Cai et al. (2023); Weber and Broström (2024)
Noise effects	Sound-sensitive species	I and O	Halvorsen et al. (2012); Pine et al. (2012); Voellmy et al. (2014); Solan et al. (2016); Gigot et al. (2023a, 2023b); Cones et al. (2024)
Changes in the composition of benthic communities	Infaunal and epifaunal communities	I and O	Wilhelmsson et al. (2006a, 2006b); Wilhelmsson and Malm (2008); Andersson and Öhman (2010); De Backer et al. (2014); Vandendriessche et al. (2015); Coates et al. (2014, 2015, 2016); Griffin et al. (2016); Krone et al., (2013a, 2017); Van Hal et al. (2017); Wang et al. (2019); Hutchison et al. (2020a); Lu et al. (2020); Causon et al. (2022); Coolen et al. (2022); Ter Hofstede et al. (2022); Lefaible et al. (2023); Li et al. (2023)
Release of contaminants	Seabed; seawater	I and O	Wang et al. (2023)
Hard-bottom habitat creation	Fouling assemblages; seabed	O	Wilhelmsson et al. (2006a, 2006b); Wilhelmsson and Malm (2008); Joschko et al. (2008); Andersson et al. (2009); Lindeboom et al. (2011); Krone et al. (2013b); De Mesel et al. (2015); Hutchison et al. (2020a); Karlsson et al. (2022); Zupan et al. (2023, 2024); Kingma et al. (2024); Ter Hofstede et al. (2024)
Colonization of non-native species	Fouling assemblages	O	Lindeboom et al. (2011); Krone et al. (2013b); De Mesel et al. (2015); Hutchison et al. (2020a); Zupan et al. (2024)
Changes in oxygen and nutrient fluxes	Ecosystem functioning	O	Janßen et al. (2015); Wang et al. (2019); Daewel et al. (2022); Mavraki et al. (2022); Voet et al. (2023); Coolen et al. (2024)
Benthic organic enrichment	Seabed; ecosystem functioning	O	Andersson and Öhman (2010); Krone et al. (2013b); Coates et al. (2014, 2015); Ivanov et al. (2021); Serpetti et al. (2021); Coolen et al. (2022); Daewel et al. (2022); Mavraki et al. (2022); Lefaible et al. (2023); Wang et al. (2023); Voet et al. (2023)
Changes in primary production	Ecosystem functioning	O	Wang et al. (2019); Daewel et al. (2022); Cai et al. (2023)
Reef effect	Epifaunal community	O	Wilhelmsson et al. (2006a, 2006b); Wilhelmsson and Malm (2008); Andersson et al. (2009); Andersson and Öhman (2010); Bergström et al. (2013); Janßen et al. (2013); Reubens et al. (2011, 2013, 2014); Vandendriessche et al. (2015); Stenberg et al. (2015); Griffin et al. (2016); Krone et al. (2013a, 2017); Van Hal et al. (2017); Wang et al. (2019); Hutchison et al. (2020a); Karlsson et al. (2022); Ter Hofstede et al. (2022); Buyse et al. (2022, 2023b); Li et al. (2023); Berges et al. (2024); Labourgade et al. (2024); Werner et al. (2024)
Refuge effect	Infaunal and epifaunal community	O	Lindeboom et al. (2011); Reubens et al. (2014); Vandendriessche et al. (2015); Stenberg et al. (2015); Coates et al. (2016); Van Hal et al. (2017); Buyse et al. (2022, 2023a, 2023b)
Effects of electromagnetic fields	Electro-sensitive species	O	Westerberg and Lagenfelt (2008); Jakubowska et al. (2019); Hutchison et al. (2020b); Scott et al. (2018, 2021)
Changes in trophic relationships	Ecosystem functioning	O	Reubens et al. (2013, 2014); Wang et al. (2019 ); Buyse et al. (2023a, 2023b)

### Conceptual ecosystem models

**Fig. 1:**
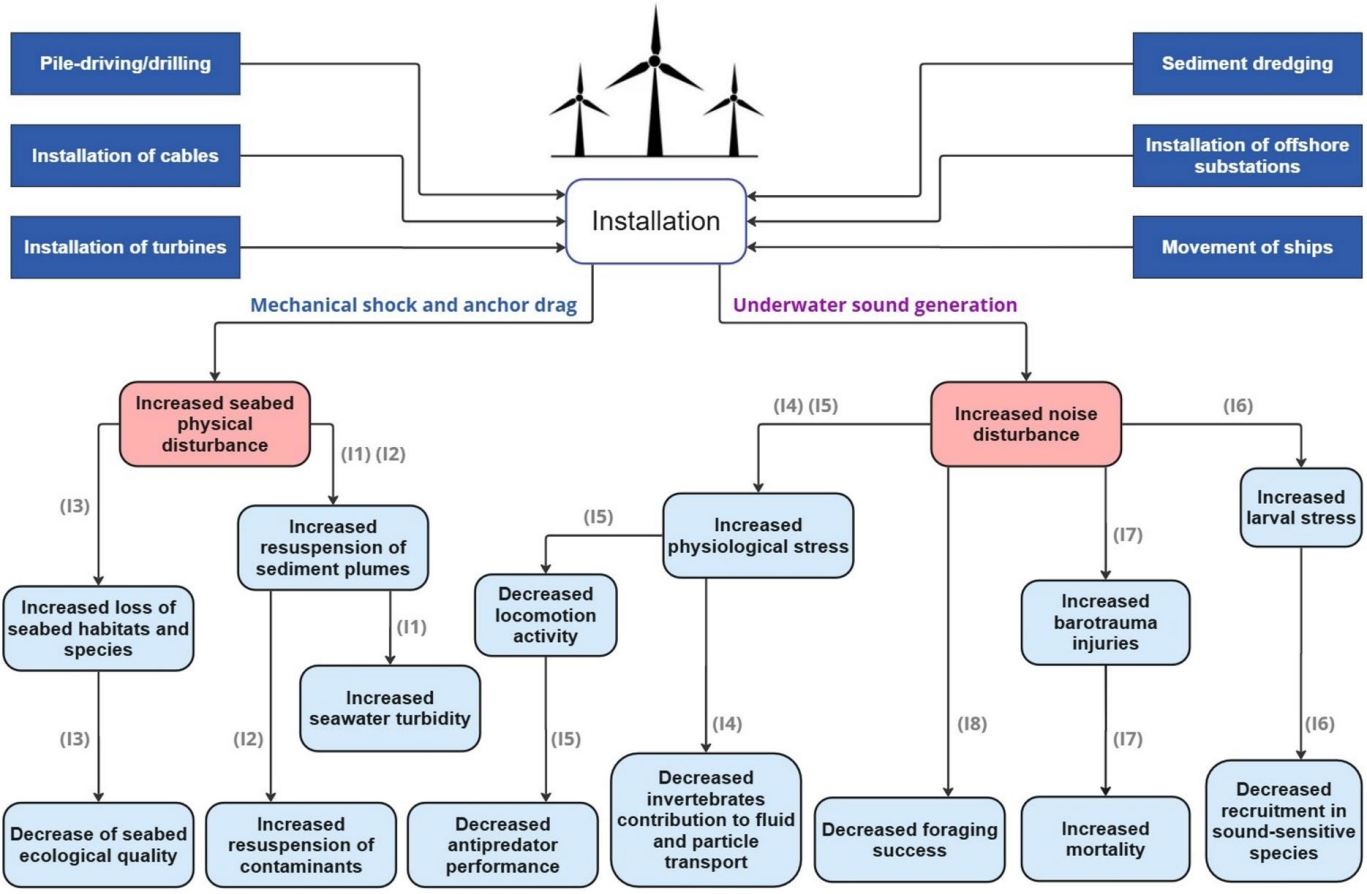
Conceptual ecosystem model illustrating the ecological-causal nexus related to the installation of offshore wind projects and their respective effects on the benthic environment and associated components. Dark blue frames indicate the installation activities (deliberate actions) and their resulting pressures (in purple font). The solid black arrows indicate the direction of the cascade effects, including direct effects (pink frames) and indirect effects (light blue frames) sequentially represented across multiple branches (ecological-causal processes). The branch IDs are shown in gray font on the arrows, numbered I1 to I8. Different branch IDs localized to the same arrow indicate branches that will split in the model’s sequence

For the installation phase, the main project activities identified were pile-driving/drilling, sediment dredging, installation of turbines, cables, and offshore substations, and the movement of ships (see details in Appendix B, supplementary material). Pressures from these project activities include seabed mechanical shock, anchor drag, and underwater sound generation. A total of eight branches were identified in the model diagram (branch IDs I1–I8; Fig. 1), derived from two direct effects.

The seabed physical disturbance is the direct effect of the mechanical shock and anchor drag, triggering indirect effects in the seawater, such as the resuspension of sediment and contaminants, and increased turbidity (I1 and I2). The indirect effects on the seabed include the loss of habitat and species, and a decrease in seabed ecological quality (I3). Noise disturbances in sound-sensitive species are a direct effect of underwater sound generation, triggering indirect effects such as barotrauma injuries, physiological stress, and larval stress, which ultimately compromise the behavior, fitness, and survival of the sensitive fauna (I4–I8, Fig. 1).

**Fig. 2:**
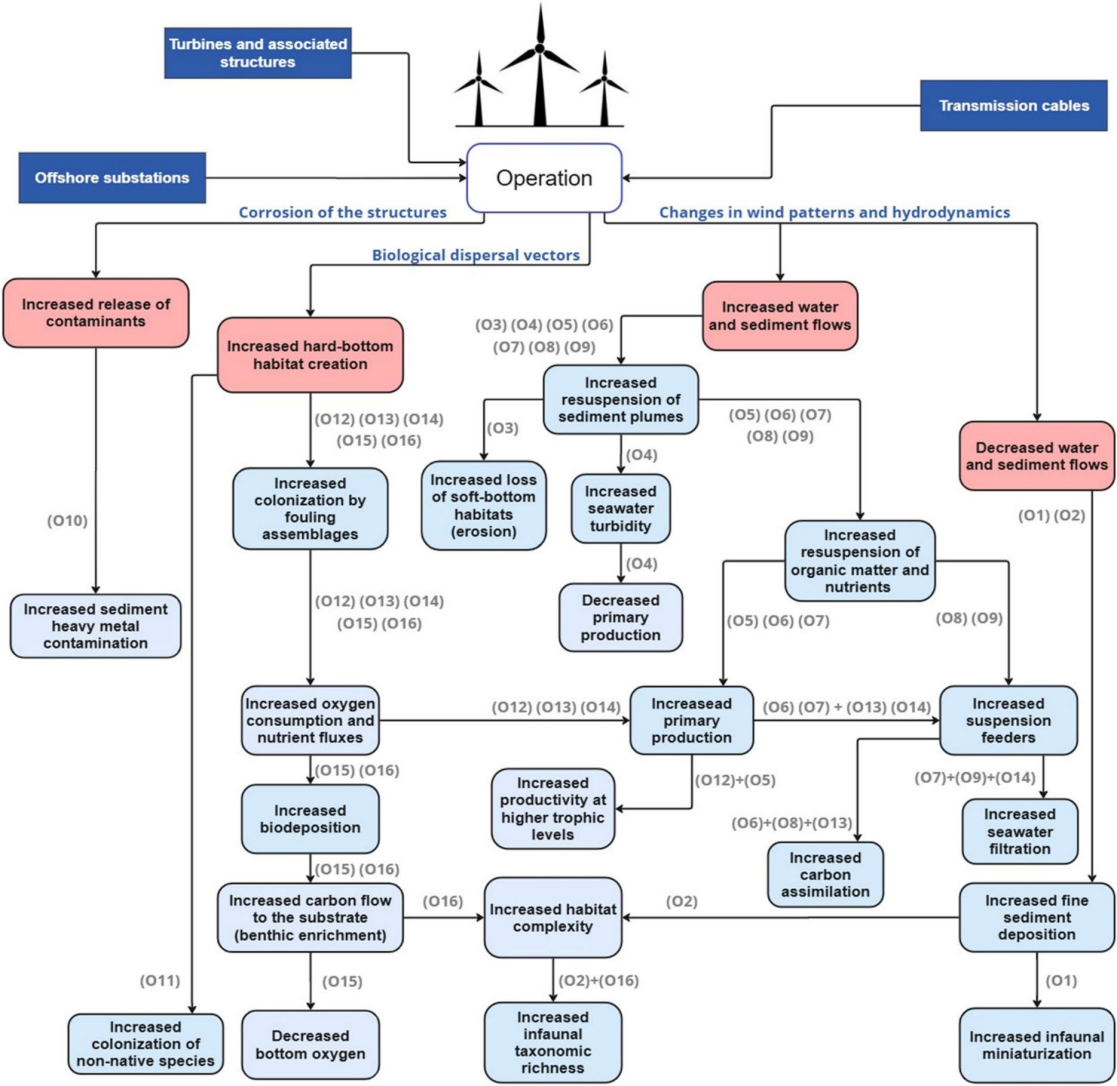
Partial conceptual ecosystem model illustrating the ecological-causal nexus related to the operation of offshore wind projects and their respective effects on the benthic environment and associated components. Dark blue frames indicate the installation activities (deliberate actions) and their resulting pressures (in purple font). The solid black arrows indicate the direction of the cascade effects, including direct effects (pink frames) and indirect effects (light blue frames) sequentially represented across multiple branches (ecological-causal processes). The branch IDs are shown in gray font on the arrows, numbered O1 to O16. Different branch IDs localized to the same arrow indicate branches that will split in the model’s sequence or indicate moments with a union of branches previously separated (when signalized with + between IDs)

**Fig. 3:**
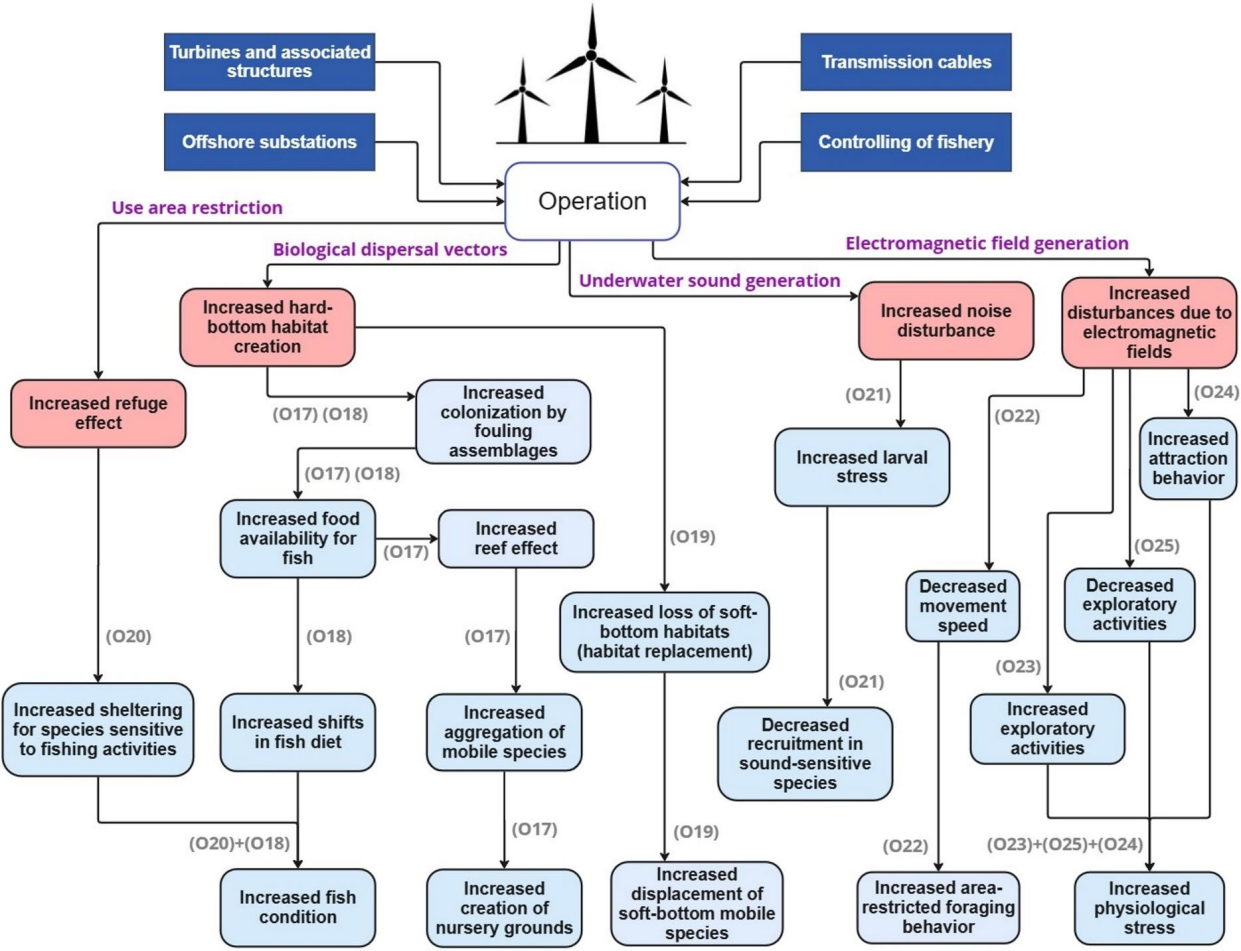
Partial conceptual ecosystem model illustrating the ecological-causal nexus related to the operation of offshore wind projects and their respective effects on the benthic environment and associated components. Dark blue frames indicate the installation activities (deliberate actions) and their resulting pressures (in purple font). The solid black arrows indicate the direction of the cascade effects, including direct effects (pink frames) and indirect effects (light blue frames) sequentially represented across multiple branches (ecological-causal processes). The branch IDs are shown in gray font on the arrows, numbered O17 to O25. Different branch IDs localized to the same arrow indicate branches that will split in the model’s sequence or indicate moments with a union of branches previously separated (when signalized with + between IDs)

Regarding the effects of the operation phase, the main identified activities of the project are the functioning of turbines and associated offshore structures, including substations and transmission cables (see details in Appendix B, supplementary material). Pressures from these activities include changes in wind patterns and hydrodynamics, which can directly affect water and sediment flows. Other pressures include corrosion of the structures (leading to the release of contaminants), underwater sound generation (related to the turbines’ operation), and electromagnetic field generation (related to the underwater transmission cables). Another evident pressure observed is the introduction of biological dispersal vectors in the marine environment associated with all installed artificial structures. This pressure promotes the direct creation of hard-bottom habitat, affecting ecosystem functioning and local benthic communities (infauna and epifauna). Lastly, the presence of the operating project also helps control fisheries (e.g., trawling), restricting area use and affecting the infaunal and epifaunal communities. In total, for the operation phase, seven direct effects were identified in the literature, deriving 25 distinct branches with indirect effects across the model (IDs from O1 to O25). To facilitate visualization of the branches, we separate the model into two diagrams (Figs. 2 and 3).

The first diagram (Fig. 2) illustrates the cascade effects of the hard-bottom habitat creation on the surrounding bottom environment, characterized by the benthic enrichment (increased carbon flow to the substrate). This process can promote increased habitat complexity and increased infaunal taxonomic richness in the surrounding sediments (O16), but can also decrease bottom oxygen (O15). The colonization of non-native species was also an indirect effect noted in relation to the creation of hard-bottom habitat (O11), and the heavy-metal contamination of the sediment is a consequence of potential corrosion of the artificial structures (O10). Also, changes in wind patterns and hydrodynamics can lead to increased or decreased water and sediment flows (Fig. 2). Increased flows promote the loss of soft-bottom habitats through erosion (O3) and reduce primary production due to intensified seawater turbidity (O4). However, the increase in primary production can occur with increased flows due to the resuspension of nutrients (O5) or due to increased nutrient fluxes associated with fouling assemblages (O12, O13, and O14). These processes can act cumulatively, improving the productivity at higher trophic levels (represented by the union of O5 and O12 in this part of the model, Fig. 2).

Additionally, suspension feeders increase with primary production, ultimately improving carbon assimilation and seawater filtration. The increase in suspension feeders and their associated processes is also driven by the resuspension of organic matter (O8 and O9) during increased-flow scenarios (Fig. 2). It means that three different processes act additively improving the carbon assimilation and seawater filtration in the model: (1) the increased organic matter resuspension (O8 and O9); (2) the primary production increased by the nutrient flux from fouling community (O13 and O14); and (3) the primary production increased by the resuspension of nutrients from the substrate (O6 and O7). Decreased water and sediment flows, in turn, generate the deposition of fine sediments. This process can favor opportunistic benthic species, thereby reducing the mean body size and biomass of infaunal communities (infaunal “miniaturization,” O1). However, fine sediment deposition can also increase habitat complexity and infaunal taxonomic richness (O2), acting additively with the benthic enrichment (branch union between O2 and O16) (Fig. 2).

The second diagram (Fig. 3) illustrates the cascade effects of the hard-bottom habitat creation on the epibenthic community, especially demersal fish and mobile invertebrates. Habitat creation allows colonization by fouling assemblages and, consequently, increases local food availability and generates the reef effect, with the aggregation of mobile species. These species can use the artificial reef as nursery grounds for reproduction (O17) or for feeding, changing their original diet (O18). The fishing prohibition in the area directly promotes the refuge effect; thus, species can also use the structures for shelter, especially those sensitive to fishing activities (O20). The refuge effect and the shifts in diet can act additively to increase the fish condition factor (Fig. 3). However, the new hard-bottom habitats, including foundations and associated structures (e.g., scour protection layers), replace the original soft-bottom habitats in the area, which can lead to the displacement of soft-bottom mobile species to other sites (O19).

This diagram (Fig. 3) shows two additional direct effects of the operation phase: increased disturbances from noise and electromagnetic fields. Noise disturbances have fewer reported effects during operation than during installation, but larval stress with consequences for recruitment in sound-sensitive species is expected (O21). Electromagnetic fields promote a series of behavioral disturbances in electro-sensitive species, including reduced movement speed and, consequently, area-restricted foraging behavior (O22). Electro-sensitive species also can increase (O23) or decrease (O25) their exploratory activities (depending on the species) and can be attracted to the cables (O24), in all cases increasing physiological stress (Fig. 3).

### Process-based causality indicators

We identified a total of 32 indicators that can be used to assess and monitor the ecological impacts of OWE projects on the benthic environment (Table 2), based on the affected parameters observed during the literature review and their connections with the ecological-causal processes organized in the ecosystem models (see Appendix C of the supplementary material for more details). More than half of the indicators (18) are useful for detecting changes in a single branch (process) of the ecosystem model. Nevertheless, 14 indicators can be used to assess effects related to two or more branches. For instance, the indicator “biomass of suspension feeders” can be used to detect changes across six branches of the model (Table 2), as this parameter is involved in multiple processes throughout the ecological-causal nexus (Fig. 2). In addition to the need to measure all indicators in the baseline phase, six indicators are important for monitoring both the installation and operation phases, four indicators are priorities only for the installation phase, and the other 22 indicators should be monitored primarily during the operation phase (Table 2).

**Table 2:** Indicators to be used for assessing and monitoring the ecological impacts of offshore wind projects on the benthic environment, highlighting the indicators’ meaning (what they represent in the ecological-causal nexus); the project phases that each indicator should be measured, including baseline (B), installation (I) and operation (O); and the branches of the conceptual ecosystem model (ecological-causal processes) that each indicator are related. Branch IDs are numbered from I1 to I8 for the installation phase and from O1 to O25 for the operation phase, following the models presented in the section above

Indicators	Indicators’ meaning	Main phases of measurement	Related model branches
Sediment mean grain size	Sediment granulometry varies with hydrodynamic changes, indicating seabed erosion (increased grain size) or deposition (decreased grain size) of fine sediments	B and O	O1, O2, and O3
Suspended sediment concentration	A proxy for increased seawater turbidity and sediment resuspension due to seabed disturbances and/or increased hydrodynamic flows	B, I, and O	I1 and O4
Sediment total organic carbon (TOC)	Signals the biodeposition and increased carbon flow to the substrate from the fouling community of the artificial structures (benthic enrichment)	B and O	O15 and O16
Amount of biofouling drop-off products (empty and full shells; biofouling debris)	Increases with biodeposition and benthic enrichment, functioning as a proxy for increased bottom habitat complexity	B and O	O16
Bottom oxygen level	Indicates a possible local anoxia/hypoxia due to the benthic enrichment and increased oxygen consumption	B and O	O15
Abundance of ecosystem engineers in the surrounding substrates	Organisms such as habitat-forming and tube-building species indicate increased soft-bottom habitat complexity and stability, and can increase especially due to the deposition of fine sediments	B and O	O2
Richness of the infaunal assemblage	Indicates increased infaunal taxonomic richness due to the increased habitat complexity	B and O	O2 and O16
Abundance of long-lived k-strategist species	Species with large body sizes and lifespans are usually sensitive to seabed disturbances. Reductions indicate decreases in local ecological quality and infaunal miniaturization	B, I, and O	I3 and O1
Abundance of opportunistic r-strategist species	Species with small lifespans and low body sizes tend to benefit from seabed disturbances. Increases indicate the reduction of local ecological quality and infaunal miniaturization	B, I, and O	I3 and O1
Total biomass of the infaunal assemblage	Decreases due to the replacement of long-lived k-strategist species by opportunistic r-strategist species, indicating reductions of local ecological quality and infaunal miniaturization	B, I, and O	I3 and O1
Heavy metal levels in the environment	Signals the release of contaminants by the corrosion of artificial structures or resuspension from sediments	B, I, and O	I2 and O10
Phytoplankton biomass	Indicates the increase or decrease of primary production	B and O	O4 and O5
Biomass of suspension feeders	Indicates changes in suspension feeders, carbon assimilation and seawater purification as a cascading result of the resuspension of organic matter, nutrients, and changes in primary production	B and O	O6, O7, O8, O9, O13, and O14
Proportion of non-native/native species in surrounding hard-bottom habitats	Indicates a possible intensified colonization of non-native species on surrounding habitats with possible negative consequences for native species	B and O	O11
Abundance of hard-bottom mobile species	Increases indicate an aggregation of mobile species related to the reef effect	B and O	O17
Abundance of soft-bottom mobile species	Decreases indicate that the replacement of soft-bottom habitats by hard-bottom habitats displaces soft-bottom species from the area. Increases indicate a general aggregation of mobile species related to the reef effect	B and O	O17 and O19
Diversity of the mobile epifaunal assemblage	Indicates if the replacement of soft-bottom habitats by hard-bottom habitats has positive or negative consequences for mobile fauna heterogeneity	B and O	O17 and O19
Residency time of fish species	Increases indicate an aggregation of fish species and an increased area use related to the reef effect	B and O	O17
Abundance of juveniles	Indicates the creation of nursery grounds for species reproduction in the project area	B and O	O17
Fish diet ratio (proportion of hard/soft-bottom prey species eaten)	A proxy for shifts in fish diet, since the availability of hard-bottom prey items tends to increase and become the dominant food resource eaten by fish	B and O	O18
Abundance of species sensitive to fishing activities	A proxy for the refuge effect and increased sheltering for species sensitive to fishing activities	B and O	O20
Mean body size of fishery species	A proxy for the refuge effect, since fishery populations can reach larger mean body sizes when sheltered against overfishing	B and O	O20
Fulton’s condition index of fish species	An integrated index to signal if the refuge effect and the shifts in fish diet (prey quality) increase or decrease the condition factor of fish species	B and O	O18 and O20
Mean time with valves fully closed for sound-sensitive bivalves	Increases indicate physiological stress due to noise disturbance, working as a proxy for reductions in invertebrates’ contribution to fluid and particle transport	B and I	I4
Mean speed of movement in sound-sensitive species	Decreases indicate physiological stress due to noise disturbance, working as a proxy for reductions in locomotion activity and antipredator performance	B and I	I5
Frequency of barotrauma injuries in fish with a swim bladder	A proxy for the increased damage to biodiversity due to noise disturbance. Fish with a swim bladder are known to be sensitive to barotrauma injuries	B and I	I7
Frequency of foraging behavior for sound-sensitive species	Indicates behavioral changes with potential decreased foraging success due to noise disturbances	B and I	I8
Abundance of sound-sensitive species	A proxy for increased mortality or reduced recruitment of sound-sensitive fauna due to noise disturbances	B, I, and O	I6, I7, and O21
Mean speed of movement in electro-sensitive species	Reductions indicate decreased movement speed, working as a proxy for increased area-restricted foraging behavior due to electromagnetic fields	B and O	O22
Depth of penetration in the sediment column for electro-sensitive species	Penetration in deeper sediment layers indicates increased exploratory activities due to electromagnetic fields, working as a proxy for increased physiological stress	B and O	O23
Time-spent ratio exploring/resting for electro-sensitive species	Disturbances of electromagnetic fields can lead electro-sensitive species to spend more time resting than exploring, which indicates increased physiological stress	B and O	O25
Abundance of electro-sensitive species	A proxy for the attraction behavior of mobile fauna due to electromagnetic fields, working as a proxy for increased physiological stress	B and O	O24

In general, sediment parameters, including data from the infaunal community, provide indicators for mostly addressing the effects of seabed disturbances, benthic enrichment, and increased habitat complexity surrounding the project. Seawater parameters, including suspended sediment concentration and phytoplankton biomass, provide useful indicators to understand changes in primary production and related trophic web consequences. Indicators from the epifaunal community, including mobile invertebrates and fish parameters, are primarily important for assessing reef and refuge effects and for addressing effects on fish diet and condition. Behavioral indicators, in turn, are especially useful to understand disturbances related to noise and electromagnetic fields (Table 2). Notably, we considered indicators of biodiversity abundance categorized by ecological groups based on life-history traits (e.g., opportunistic r-strategist species and soft-bottom mobile species) or by specific sensitivity to a pressure (e.g., sound-sensitive species and electro-sensitive species), which improves the causal meaning of the parameter within the ecological-causal nexus.

**Fig. 4:**
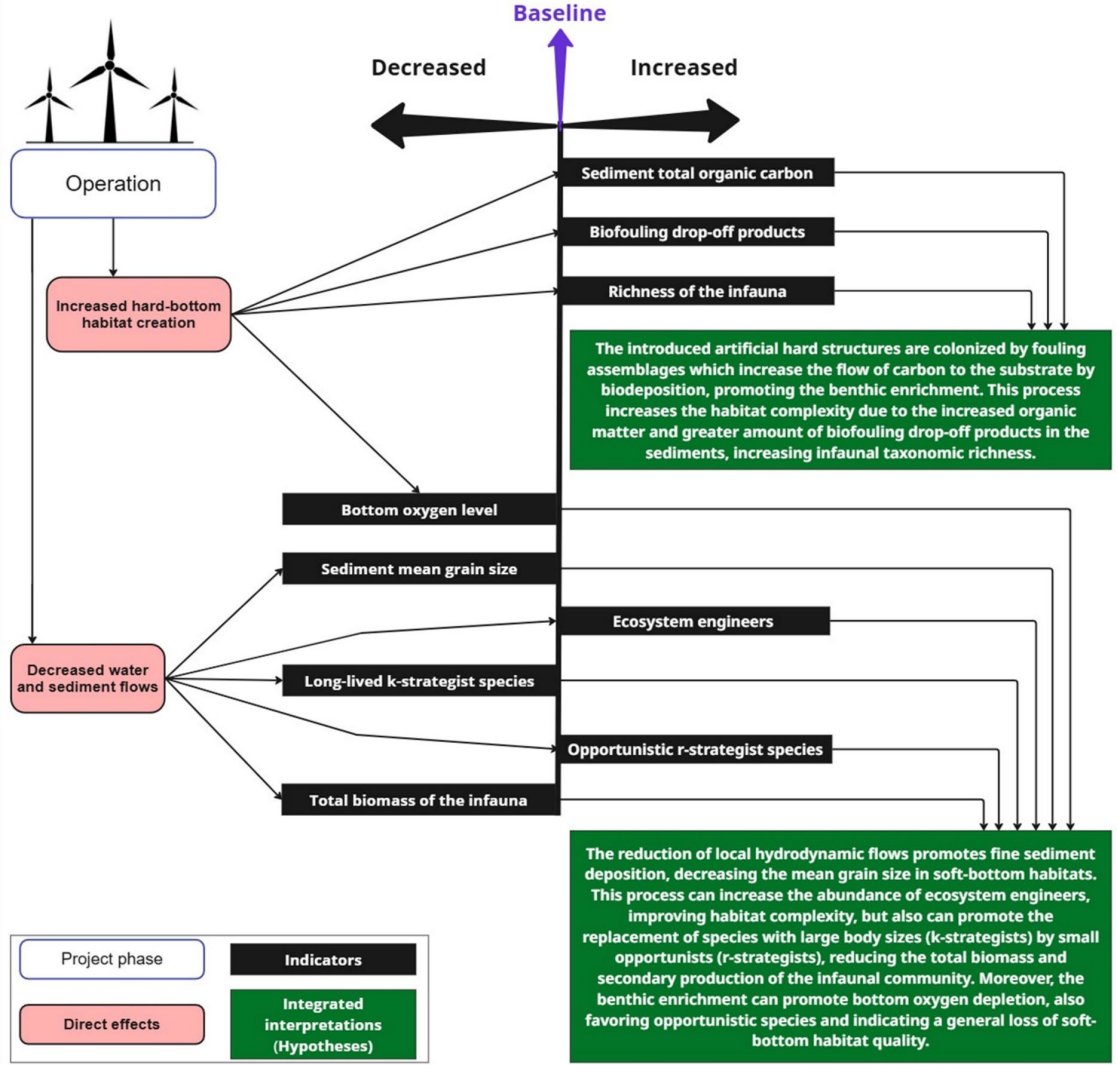
Integrated response scenarios of the indicators related to the infaunal community, proposed to assess and monitor the impacts of offshore wind energy in benthic environments during the operation phase. In this case, nine indicators are proposed to assess the positive and negative impacts of the habitat creation and decreased water and sediment flows on the infaunal community. Indicators’ responses are represented by an increase (black rectangles to the right of the indicated baseline) or a decrease (black rectangles to the left of the indicated baseline). Based on the indicators’ responses, integrated interpretations (hypotheses) of the ecological effects are proposed (green rectangles)

**Fig. 5:**
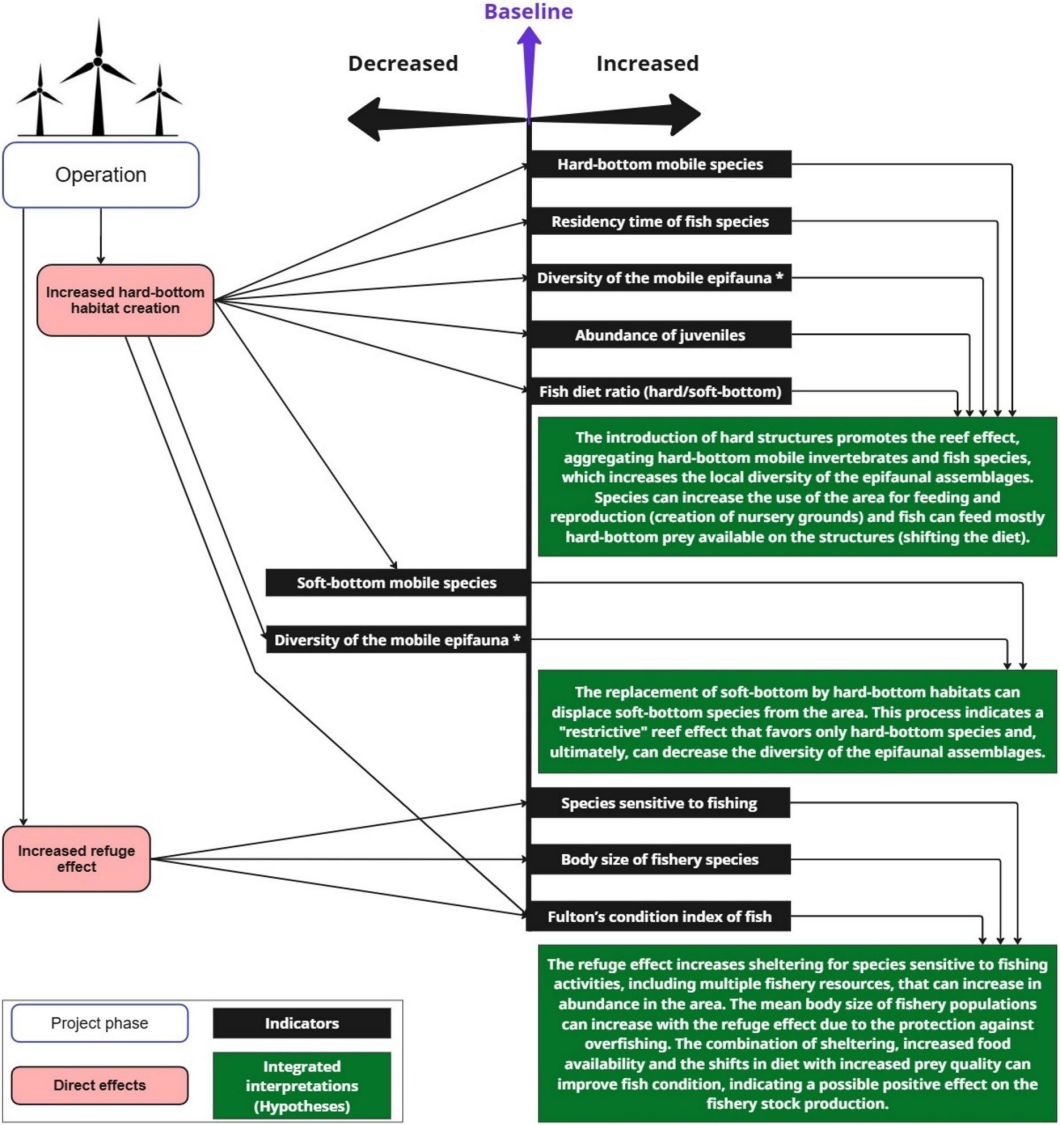
Integrated response scenarios of the indicators related to the epifaunal community, proposed to assess and monitor the impacts of offshore wind energy in benthic environments during the operation phase. In this case, ten indicators are proposed to assess the positive and negative impacts of the habitat creation and the refuge effect on the epifaunal community. Indicators’ responses are represented by an increase (black rectangles to the right of the indicated baseline) or a decrease (black rectangles to the left of the indicated baseline). Indicators marked with an asterisk (*) can increase or decrease depending on the situation. Based on the indicators’ responses, integrated interpretations (hypotheses) of ecological effects are proposed (green rectangles)

Based on evidence from these indicators, extracted from the analyzed literature, distinct hypotheses about effects can be formulated when monitoring OWE projects using integrated response scenarios (Figs. 4 and 5). For the infaunal community, nine of the proposed indicators are useful for an integrated understanding of the potential positive and negative consequences of habitat creation and decreased water and sediment flows (Fig. 4). The simultaneous increase in organic carbon, the amount of biofouling products, and species richness in the sediment would support the hypothesis that habitat creation, followed by benthic enrichment, has positive ecosystem effects related to increased soft-bottom habitat complexity (Fig. 4). However, bottom oxygen levels should also be assessed, as benthic enrichment is expected to promote oxygen depletion, a negative impact. With decreased water and sediment flows, fine sediment deposition will reduce bottom grain sizes, and the positive effects of increased habitat complexity can be improved, signaled by the increased abundance of ecosystem engineers. The negative impacts, in turn, can also be intensified if the fine sediments reduce habitat quality, triggering the replacement of species with large body sizes (k-strategists) by small opportunists (r-strategists), ultimately reducing the total infaunal biomass (infaunal “miniaturization”) (Fig. 4).

For the epifaunal community, including mobile invertebrates and demersal fish, ten of the proposed indicators are useful for an integrated understanding of the potential positive and negative consequences of habitat creation and the refuge effect (Fig. 5). The simultaneous increases in the abundance of hard-bottom mobile species, fish residency time, and species diversity would support the hypothesis that habitat creation, followed by the reef effect, has positive impacts on local biodiversity aggregation (Fig. 5). The abundance of juveniles would also signal that species are using the area as nursery grounds, while the increased presence of hard-bottom species in the fish diet would suggest its use for feeding. However, replacing soft-bottom habitats with hard-bottom habitats can displace soft-bottom species from the area, potentially reducing epifaunal diversity. The positive impacts of the reef effect can be intensified by the refuge effect, increasing shelter against fishing. This effect is signaled by increased abundance in species sensitive to fishing and by a larger mean body size among fishery resources, indicating a reduction in overfishing pressure in the area. Fishery stock production can be improved by these positive changes, which could be suggested by increases in Fulton’s condition index (Fig. 5).

## Discussion

In this study, we reviewed prior evidence on the positive and negative effects of offshore wind energy (OWE) on benthic environments, aiming to propose process-based causality indicators for baseline assessment and monitoring, with Ecosystem-Based Management (EBM) in mind. We showed that current knowledge can inform the design of evidence-based conceptual ecosystem models that qualitatively support a comprehensive understanding of the ecological-causal nexus linking project pressures to cascading expected changes across multiple benthic components and ecosystemic processes. This objective and streamlined approach allowed us to organize the existing knowledge to suggest potential parameters that could be used as indicators of the ecological-causal process, which can guide the Environmental Impact Assessment (EIA) from a “data-rich information-poor” scenario (Wilding et al., 2017) to a process-based praxis closer to the principles of EBM (Long et al., 2015). Such a tool is feasible for practical application during scoping of baseline fieldwork and can significantly reduce unnecessary costs by preventing the collection of non-meaningful data that compromises EIA efficiency. Undoubtedly, research gaps still exist, limiting model construction and indicator selection, and the applicability of this approach in a local context would raise additional challenges, which are discussed below.

### Bibliographic review: research strengths and gaps

Our literature review aimed to highlight the major potential effects of OWE projects on the benthic environment. The results of our search illustrate an imbalance in the amount of information across the different phases of the project and in the distinct general themes regarding ecological effects. The majority of the articles found presented evidence of impacts during project operation, with fewer studies evaluating impacts during the installation phase. Most studies addressing installation effects using field data refer to seabed disturbances (e.g., Coates et al., 2015; Guarinello & Carey, 2022), while the impacts related to noise mostly come from experiments, especially laboratory simulations (e.g., Cones et al., 2024; Gigot et al., 2023a, 2023b). This fact shows that field studies monitoring the impacts of the installation and comparing them with baseline data are still scarce and are needed to further refine the conceptual models and indicator selection.

Regarding the impacts of the project operation, some themes have already been extensively explored in the literature analyzed, such as the reef effect (Werner et al., 2024; Wilhelmsson et al., 2006a), the refuge effect (Buyse et al., 2022; Vandendriessche et al., 2015), and the benthic enrichment and associated changes in community composition (Coates et al., 2014; Lefaible et al., 2023). Thus, the indicators selected for assessing and monitoring impacts within these themes are the most robust. On the other hand, there is little evidence on topics such as the colonization of non-native species. Studies addressing this theme were mostly developed in countries with temperate climates (De Mesel et al., 2015; Zupan et al., 2024). For countries with tropical climates, such as some South American countries, where offshore wind energy is about to be developed, this issue is a crucial concern, since such regions are typically prone to biological invasions (e.g., Soares et al., 2022).

The understanding of changes in ecosystem functions, including primary production and the trophic web, was also addressed by a few studies. The majority of the articles on this theme used modeling approaches (Daewel et al., 2022; Wang et al., 2019), and others directly assessed fish diet (Buyse et al., 2023a; Reubens et al., 2014). Both techniques yield valuable information for proposing indicators but still represent evidence restricted to particular regions or species. Another important gap is that, although some papers addressed ecosystem functions as mentioned above, the ecosystem services related to the benthic components were not present as the focus of any case study evaluated. Such a gap illustrates a crucial limitation toward a comprehensive understanding of the benthic environment and a current barrier to promoting EBM principles into the EIA (Bravo et al., 2023; Laurino et al., 2025). Additionally, the increased heavy metal contamination due to structural corrosion was the least studied theme, represented by a single article (Wang et al., 2023). Lastly, the effects of electromagnetic fields, as observed for noise effects, are primarily based on laboratory simulations (Hutchison et al., 2020b; Scott et al., 2018, 2021), and the response of the proposed indicator in a field context is still poorly understood. Thus, we suggest that the themes highlighted above require further study to improve EIA in future projects.

### Conceptual ecosystem models: potentialities and limitations

Our conceptual ecosystem models aimed, in the simplest way possible, to highlight the cascade of expected changes resulting from OWE projects’ impacts on the benthic environment, providing a logical platform for interpreting the results of the ecological-causal process. We showed that qualitative diagrams, supported by prior evidence, can predict causal relationships, component responses (increases or decreases), and cascade indirect effects (Perdicoúlis & Glasson, 2006; Perdicoúlis & Piper, 2008). Additionally, the ecological-causal processes identified across the branches of the diagrams can provide insights into cumulative effects (Dibo et al., 2018; Perdicoúlis & Piper, 2008), especially because branch unions symbolize additive processes that converge on the same ecological consequence. Our models showed several examples of this, such as the additive effects of benthic enrichment and fine-sediment deposition on increasing habitat complexity (see Lefaible et al., 2023), and the additive effects of diet shifts and refuge on improving fish condition (see Reubens et al., 2014). Addressing cumulative effects is an EBM principle (Long et al., 2015) and has been growing in the context of wind energy (e.g., Declerck et al., 2023; Gușatu et al., 2021; Kuempel et al., 2025; Willsteed et al., 2023), including the synergism with external pressures such as climate change (Nogues et al., 2022, 2023; Trifonova & Scott, 2023; Voet et al., 2022). Our models are therefore examples that can be complemented with threats from other drivers, aiming to raise hypotheses about the cumulative effects of OWE.

The use of different case studies allowed us to note that particular changes can follow distinct pathways (sometimes opposed) in the model, suggesting the need for attention concerning project-dependent effects. Hydrodynamic changes, for instance, can be characterized by increased flows (e.g., Weber & Broström, 2024), but there is also evidence that OWE farms can reduce local hydrodynamic flows (e.g., Daewel et al., 2022). The direction, intensity, and consequences of these changes seem to depend on project features such as foundation type (e.g., jacket, monopile) and position concerning local currents (Cai et al., 2023; Coates et al., 2014; Ivanov et al., 2021; Lefaible et al., 2023). In scenarios with increased hydrodynamics, primary production can decrease due to sediment resuspension and increased turbidity, but can also increase due to resuspension of bottom nutrients, which seems to be a site-dependent process (Daewel et al., 2022; Vanhellemont & Ruddick, 2014; Wang et al., 2019). In fact, predicting a general pattern of change for primary production linked to benthic disturbances is challenging, especially because, in addition, suspension feeders tend to increase with primary production by a bottom-up process (Wang et al., 2019). In turn, these organisms consume phytoplankton and ultimately exert top-down control over primary production (Joschko et al., 2008). In addition to these project-dependent and site-dependent effects, we also noted species-dependent effects that generate distinct pathways within the models. These cases were mostly related to the effects of electromagnetic fields, which can distinctly affect different species (Hutchison et al., 2020b; Scott et al., 2018, 2021). Therefore, our models showed a general ecological-causal nexus that, for local application, needs to incorporate particular context elements.

It is also important to highlight that the causal processes discussed operate at distinct temporal and spatial scales, meaning that different model pathways can signal scale-dependent outcomes. Considering the appropriate scales of processes is also a crucial EBM principle (Long et al., 2015), which can help not only with the interpretation of models but also with determining the appropriate study area for each proposed indicator (Laurino et al., 2025). For instance, the artificial reef effect and benthic organic enrichment operate locally, within a few meters of the turbines and other hard structures, usually producing small-scale outcomes (e.g., Lefaible et al., 2023). On the other hand, changes in primary production can span large spatial scales, depending on external factors such as the cumulative effects of localized impacts across projects, underscoring the importance of surrounding regional features for evaluation (Daewel et al., 2022). As another example, the substitution of soft-bottom habitats by artificial hard structures, with the displacement of soft-bottom species, should be considered relative to the surrounding regional environmental composition, since such an effect may be a relatively minor concern in a mostly homogeneous soft-sediment region. Therefore, although several effects of OWE have small-scale outcomes when analyzed at the project level, regional features and potential cumulative effects must be considered to achieve a comprehensive understanding of their consequences, helping governments, stakeholders, and project proponents to weigh the importance of the multiple indicators.

Our models were based exclusively on evidence from the literature, considering original case studies related to OWE projects and benthic environments; thus, they leave out branches or effect chains that may be clearer in other settings. Consequently, some causal processes (model branches) ended where no additional consequences were evidenced, although we can deductively suggest some. For instance, increased sediment resuspension is expected during installation and operation (Vanhellemont & Ruddick, 2014; Huang, 2022; Cai et al., 2023). This change ultimately influences local suspension feeders and associated processes (e.g., seawater filtration). Although we found studies corroborating this consequence only for the operation phase (Joschko et al., 2008; Voet et al., 2023; Wang et al., 2019), we can hypothesize that it would also be present during the installation phase. As another example, decreased bottom-water oxygen levels have already been documented in OWE projects (Daewel et al., 2022; Janßen et al., 2015), but the consequences of these changes have not yet been thoroughly investigated. Nonetheless, studies evaluating sediment entrapment of organic material under severe oxygen depletion in other contexts indicate that such changes can be accompanied by the release of hydrogen sulfide (H_2_S), which can promote faunal mortality (Powilleit & Kube, 1999). This illustrates how our models can be further expanded as hypotheses like these are future tested within the specific context of OWE projects.

In a practical context, the models can also be used for gap analysis in specific regions where OWE projects are planned, such as Brazil (Hernandez et al., 2021; Laurino et al., 2025). In these cases, hypothetical causal processes could first be assessed using a “brainstorming” conceptual-qualitative model based on different local knowledges of the local processes (Turra et al., 2017). Workshops that encourage stakeholder participation can bring together scientists, fishers, OWE developers, and consultants to generate hypotheses about the causal processes underlying the development of OWE projects in a given area. In this context, the evidence-based models derived from our results could be used to determine which brainstorming hypotheses raised by stakeholders have already been evidenced elsewhere, and which need further research before OWE development in the area. In addition to addressing research gaps, the development of these brainstorming models builds a platform for the participation and use of diverse knowledge systems in impact assessment, which also aligns with EBM principles (Andrade & Turra, 2021; Long et al., 2015). Therefore, we suggest that combining evidence-based models, such as those developed in the present study, with brainstorming multi-knowledge models is the best way to achieve a comprehensive understanding of the ecological-causal processes associated with OWE projects in a given area. Although here we focused on the benthic environment, such a strategy could be further expanded for other vulnerable components, including marine mammals, sea turtles, and seabirds, ensuring an integrated ecosystemic view of the OWE causal processes.

### Process-based causality indicators as a tool to improve EIA

We proposed a set of parameters that can serve as indicators during the EIA process for OWE projects and, based on the nexus of the conceptual models, can represent the ecological-causal process. Some of the suggested parameters are relatively obvious and directly related to specific changes. They include bottom oxygen levels (assessing oxygen depletion), heavy metal levels (assessing contaminant release), and the proportion of non-native to native species (assessing biological invasion). However, some of them needed a more holistic view of the causal nexus to be accurately identified, as is the case with the abundance indicators. It would be difficult to understand the causal processes and ecosystem consequences of changes in infauna and epifauna if we only consider changes in the total community’s abundance. For example, benthic enrichment is often associated with an overall increase in infaunal community abundance (Coates et al., 2014; Coolen et al., 2022; Lefaible et al., 2023), suggesting a positive impact. On the other hand, when we examine this variation in abundance separately across distinct ecological groups, we find that it may reflect an increase in opportunistic species (Lu et al., 2020), indicating reduced local habitat quality. The same is true for mobile epifauna; if we only consider total abundance, we will usually see an overall increase in aggregation (Wilhelmsson et al., 2006a, 2006b; Andersson and Öhman, 2010; Karlsson et al., 2022), while if we classify the community into groups such as hard-bottom and soft-bottom species, we may notice a possible restrictive characteristic of the reef effect (Griffin et al., 2016; Krone et al., 2017; Van Hal et al., 2017). Besides the turbine foundations, the scour protection layers also added more hard structures (rocks) over the sediments, intensifying the loss of soft-bottom habitats and usually favoring the aggregation of hard-bottom species (Krone et al., 2017). Considering groups according to their sensitivity to different project-related pressures (i.e., sound-sensitive, electro-sensitive, fishing-sensitive) is also essential for identifying the causes of each change in abundance or behavior (Gigot et al., 2023a, 2023b; Scott et al., 2018, 2021). Therefore, the causal interpretation of the abundance indicators improves when we consider organisms grouped by their life-history traits.

The strategy of determining indicators based on ecological groups and traits is not new and has been explored for different contexts of impact assessment. The review performed by Taormina et al. (2022) presented examples of trait-based indicators used to evaluate ecological changes caused by artificial structures in marine environments, although the authors note that this approach is less common than the use of traditional indicators (such as total community abundance and density). Trait-based indicators are also used to construct multimetric indices that integrate parameters from distinct ecological groups (e.g., opportunists, tolerant, and sensitive) into a single numerical value (Borja et al., 2000; Taormina et al., 2022). In the specific context of OWE projects, such approaches are still incipient, with recent good examples of indicators to assess and monitor fishery resources, macrobenthos, and ecosystem functioning (Lefaible et al., 2025; Methratta, 2024; Trifonova & Scott, 2023), including parameters also proposed in our results (e.g., fish condition, diet, and trait-based metrics). Functional richness and diversity, trait-based indices, were also already proposed, revealing that community values for this indicator can increase close to OWE projects (Lefaible et al., 2025) and can be similar to those of natural hard-bottom habitats in the short term (during early colonization), although values can decrease in the long term (climax stage) (Boutin et al., 2023).

The assessment of trait-based and functional indicators has also been improving with the use of advanced sampling tools in the offshore wind sector. For instance, optical tools such as the Sediment Profile Imagery (SPI) are used to interpret the status and function of infaunal benthic communities, generating valuable results without the need for destructive sampling devices (e.g., grabs, cores) to assess taxonomic composition (Solan et al., 2003). SPI captures organism-sediment relationships in situ and provides trait information on the functional benthic community (e.g., opportunists), as well as on biogeochemical processes (e.g., sediment oxygen demand), and has been extensively used for impact assessment and monitoring (Germano et al., 2011). In the offshore wind sector, specifically, there are several examples of projects from the USA that use SPI to assess benthic traits and functional indicators (e.g., South Fork Wind, 2020; Revolution Wind, 2023). Despite these advances, official guidelines and research protocols for assessing and monitoring the impacts of renewable energy development, including wind farms, rarely recommend using trait-based indicators with causal meaning (Laurino et al., 2025). The guideline protocols developed by the Bureau of Ocean Energy Management (USA) are examples of that (BOEM, 2019, 2023). Guidelines on fish indicators are largely based on total-community or species-centered information (BOEM, 2023), and for benthic habitats, no indicators are explicitly proposed (BOEM, 2019). In this context, our results can contribute to improving future protocols toward a trait-based approach, although it depends on developing direct communication channels between scientists, government agencies, and project developers to discuss and incorporate these advances.

The indicators proposed here can reflect changes in a set of ecological processes, leading to hypotheses about ecological consequences that ultimately support the assessment of OWE impacts on the provision of marine ecosystem services, another aspect related to EBM. Examples of supporting services (ecological functions) highlighted by Le et al. (2017) that could be related to the indicators here proposed include primary production (phytoplankton biomass), secondary production (infaunal biomass), creation of nursery habitats (abundance of juveniles), and bioturbation (depth of penetration in the sediment column). Proxies for provisioning services can be associated with fishery resources indicators, such as Fulton’s condition index, which would signal a possible increase in fishery stock production and quality (Buyse et al., 2023a; Reubens et al., 2014). Regulating services, in turn, can be represented by the biomass of suspension feeders, which is indirectly associated with biological filtration (seawater purification) and carbon assimilation (carbon sequestration and climate regulation) (Mavraki et al., 2022; Voet et al., 2023; Watson et al., 2024). Incorporating an ecosystem-services approach into impact assessment is a recommended strategy toward an EBM perspective and is growing rapidly in the context of OWE (Baulaz et al., 2023; Bravo et al., 2023; Causon & Gill, 2018; Watson et al., 2024). However, this requires an in-depth study of ecosystem services and an understanding of how each project activity can directly and indirectly affect them. As a next step, it is important to expand these associations by systematically proposing feasible indicators of impacts for multiple ecosystem services (e.g., Van de Pol et al., 2023), based on previous studies that have already surveyed these impacts in the context of OWE projects (e.g., Baulaz et al., 2023; Watson et al., 2024). These approaches can also include cultural services associated with the benthic environment, such as recreational diving (e.g., Niz et al., 2023), thereby increasingly integrating the ecological and socioeconomic dimensions into the causal nexus of impacts.

## Conclusions

This study proposes a set of indicators to describe the baseline and monitor the impacts of offshore wind energy on benthic environments and explores a general, theoretical application of the concept of process-based causality indicators. The proposed indicators are grounded in causal processes and can be used in practice to detect ecological changes, ultimately enhancing the effectiveness of impact assessments for offshore wind energy development. Additionally, we developed conceptual models that are both highly applicable and easy to implement, while also identifying gaps for future research. Nevertheless, as we introduced a theoretical approach, future analyses are still needed to test its practical implementation in real-world cases within specific local contexts. The definition of indicators based on the qualitative understanding of the causal nexus, here focused on the benthic environment, can serve as an exemplary approach that could be replicated for other components of the marine environment, such as mammals, birds, and sea turtles. Furthermore, the conceptual models can be expanded in the future to include ecosystem services and their benefits to people, thereby improving visualization of the connections between the ecological and socioeconomic dimensions within the causal nexus, which could then be evaluated through socioecological indicators.

A proposal for comprehensive process-based indicators is a step toward rethinking EIA under a more holistic and integrated perspective, which is urgently needed in the face of planetary crises. These and other tools anchored in EBM principles can be an appropriate approach for an EIA process that considers the impacts of the offshore industry on natural systems, contributing to efficient solutions to mitigate harmful impacts and maximize positive outcomes. In this context, our results provide significant insights that can already be applied to new OWE projects currently in the planning phase. Our proposed trait-based indicators serve as a useful tool in this context. For local applications, OWE developers, consultancies, and environmental agencies can utilize these indicators, presented here in a general format, and adapt them to specific contexts and scales. We therefore suggest that, before conducting baseline fieldwork, the proposed indicators, especially those related to organisms within the different ecological groups, be locally identified (including opportunists, k-strategists, sound-sensitive, electro-sensitive, fisheries-sensitive, and ecosystem engineers). Once identified, the abundance reference values of such indicators can be measured during the baseline phase and subsequently monitored to detect changes in the local context. The appropriate detection of changes is fundamental to proposing management actions to mitigate impacts and ultimately to ensuring that wind energy development meets its commitment to sustainability.

## Supplementary Information

Below is the link to the electronic supplementary material.

ESM 1 (XLSX 39.4 KB)

## Data Availability

No datasets were generated or analysed during the current study.
